# A new antagonist for CCR4 attenuates allergic lung inflammation in a mouse model of asthma

**DOI:** 10.1038/s41598-017-11868-9

**Published:** 2017-11-08

**Authors:** Yang Zhang, Yinfang Wu, Hui Qi, Junhai Xiao, Hongwei Gong, Yan Zhang, Enquan Xu, Song Li, Dalong Ma, Ying Wang, Wen Li, Huahao Shen

**Affiliations:** 1 Department of Immunology, School of Basic Medical Sciences, Key Laboratory of Immunology, Ministry of Health, Peking University Health Science Center, Beijing, 100191 China; 2 Laboratory of Computer-Aided Drug Design & Discovery, State Key Laboratory of Toxicology and Medical Countermeasures, Beijing Institute of Pharmacology and Toxicology, Beijing, 100850 China; 3 Department of Respiratory Medicine, the Second Affiliated Hospital School of Medicine of Zhejiang University, Zhejiang University institute of Respiratory Diseases, Hangzhou, 310009 China; 4 School of Pharmaceutical Sciences, Jilin University, Changchun, 130021 China; 5 Department of Hematology, Peking University First Hospital, Beijing, 100034 China; 6 Beijing Children’s Hospital, Capital Medical University, Beijing, 100045 China

## Abstract

CCR4 is highly expressed on Th2 cells. CCR4 ligands include CCL22 and CCL17. Chemokine-like factor 1 can also mediate chemotaxis via CCR4. We designed and synthetized novel CCR4 antagonists, which were piperazinyl pyridine derivatives, for disrupting the interaction between three ligands and CCR4. We also determined whether these novel CCR4 antagonists could alleviate allergic asthma in a mouse. For identifying the potent compounds in *vitro*, we used chemotaxis inhibition and competition binding assays induced by CCL22, CCL17 and one of CKLF1’s C-terminal peptides, C27. We found compound 8a which showed excellent potency in blocking the interaction of CCR4 and its three ligands. For studying the specificity of compounds, we chose chemotaxis inhibition assays with different receptors and ligands. We found compound 8a had excellent receptor specificity and exerted few influence on the interaction of other receptors and their ligands. In the 3-(4,5-dimethylthiazol-2-yl)-2,5-diphenyltetrazolium bromide assay, compound 8a had no obvious cytotoxicity till the higher concentration (16 μM). For determining the potency of compounds in blocking the interaction of CCR4 in *vivo*, we used the ovalbumin induced allergic asthma model in mice. Our study demonstrated that CCR4 blockaded by compound 8a effectively attenuated airway hyperresponsiveness, airway eosinophilia and Th2 cytokines.

## Introduction

Allergic asthma is a chronic inflammatory disorder characterized by the infiltration of inflammatory cells, such as Th2 cells, eosinophils and mast cells, into the lungs and airways^1^. Th2 T cells play a central role in airway inflammatory response by regulating IgE production, accumulation, and activation of eosinophils, as well as contributing to airway remodeling involving the epithelium and fibroblasts. The accumulation of Th2 T cells in the lungs is essential for both the initiation and persistence of airway inflammation, and studies in asthmatic volunteers have shown marked increases in Th2 T cells in the lungs after in *vivo* allergen challenge. Chemokine receptor 4 (CCR4) is highly expressed on Th2 cells and plays a key role in Th2 T cell recruitment into the asthmatic airways^2–5^.

Thymus and activation-regulated chemokine (TARC)/CCL17^6^ and macrophage-derived chemokine (MDC)/CCL22^7^ are known ligands of CCR4. CCL17 and CCL22 are up-regulated in the lungs of patients with allergic asthma^8,9^. Like the CCR4 antibody, the special antibodies against CCL17 and CCL22 can also reduce airway eosinophilia and hyperresponsiveness in asthmatic mice elicited by ovalbumin (OVA)^10,11^. Therefore, CCR4 and its ligands (CCL17 and CCL22) play important roles in asthmatic inflammations.

Chemokine-like factor1 (CKLF1) also uses CCR4 as functional receptor^12^. CKLF1 does not possess the traditional structure of classical chemokines but exhibits chemotactic activity on a broad spectrum of leukocytes^13^. CKLF1 is highly expressed on the bronchial mucous membrane of asthma patients. Mice with overexpressed CKLF1 have significant pathological changes that are similar to those of asthma, such as airway remodeling, peribronchial leukocyte infiltration in addition to epithelial shedding, collagen deposition, inflammatory exudates in the lumen^14^. Similar and obvious changes were also taken place in the lungs of CKLF1-transgenetic mice (unpublished data). Further studies show that CKLF1 C-terminal peptides C19 can inhibit cell chemotaxis induced by CKLF1, CCL17 and CCL11 in *vitro* and reduce airway eosinophilia, lung inflammation, and airway hyperresponsiveness in the asthmatic mouse model^15,16^.

Corticosteroids and long-acting beta2-agonists is a common approach to control asthma symptoms and prevent acute exacerbations, but their drug resistance and side-effects desire novel therapeutic strategies. Therefore, antagonists targeting the interaction of CCR4 and their ligands could be attractive medicines against allergic asthma by inhibiting Th2 cell migration to inflammatory sites.

A series of small molecular CCR4 antagonist classes have been discovered^17–24^. Compound 22 is a highly active CCR4 antagonist in the reported compounds^17,25^. All of the CCR4 antagonists above are inhibitors of the interaction of CCR4 and CCL22 or CCL17. In order to develop more valid CCR4 antagonists, a series of piperazine pyrimidine derivatives were designed and synthesized based on the interaction of CCR4 with CKLF1 and the structure activity relationship of compound 22^25^. The activities of all the newly designed and synthesized compounds were evaluated using a chemotaxis assay. Among them, 1 μM compound 8a blocked CCL22 or CCL17 mediated chemotaxis was similar to compound 22. However, compound 8a exerted a more positive inhibition of chemotaxis mediated by C27 than compound 22.

For exploring therapeutic potential of compound 8a as a drug used to treat allergic asthma, in this study, we assessed effective and specific activity of compound 8a targeting the interaction of CCR4 and their ligands and its toxicity in *vitro*. Further, we demonstrated in *vivo* effectiveness of compound 8a in a murine model of allergic asthma.

## Results

### Activity of compound 8a

**Figure 1:**
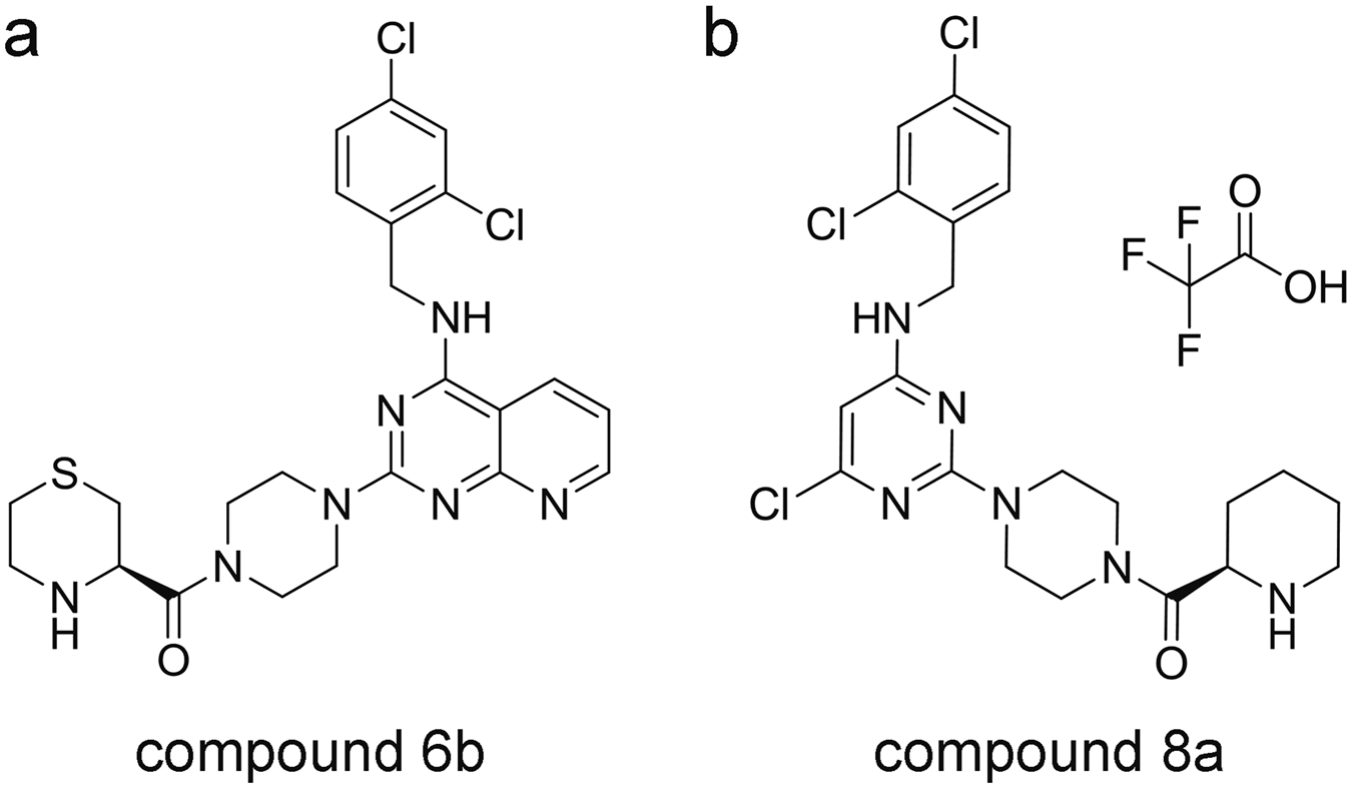
The structure of compound 6b and compound 8a. (**a**) Compound 6b. (**b**) Compound 8a.

**Figure 2:**
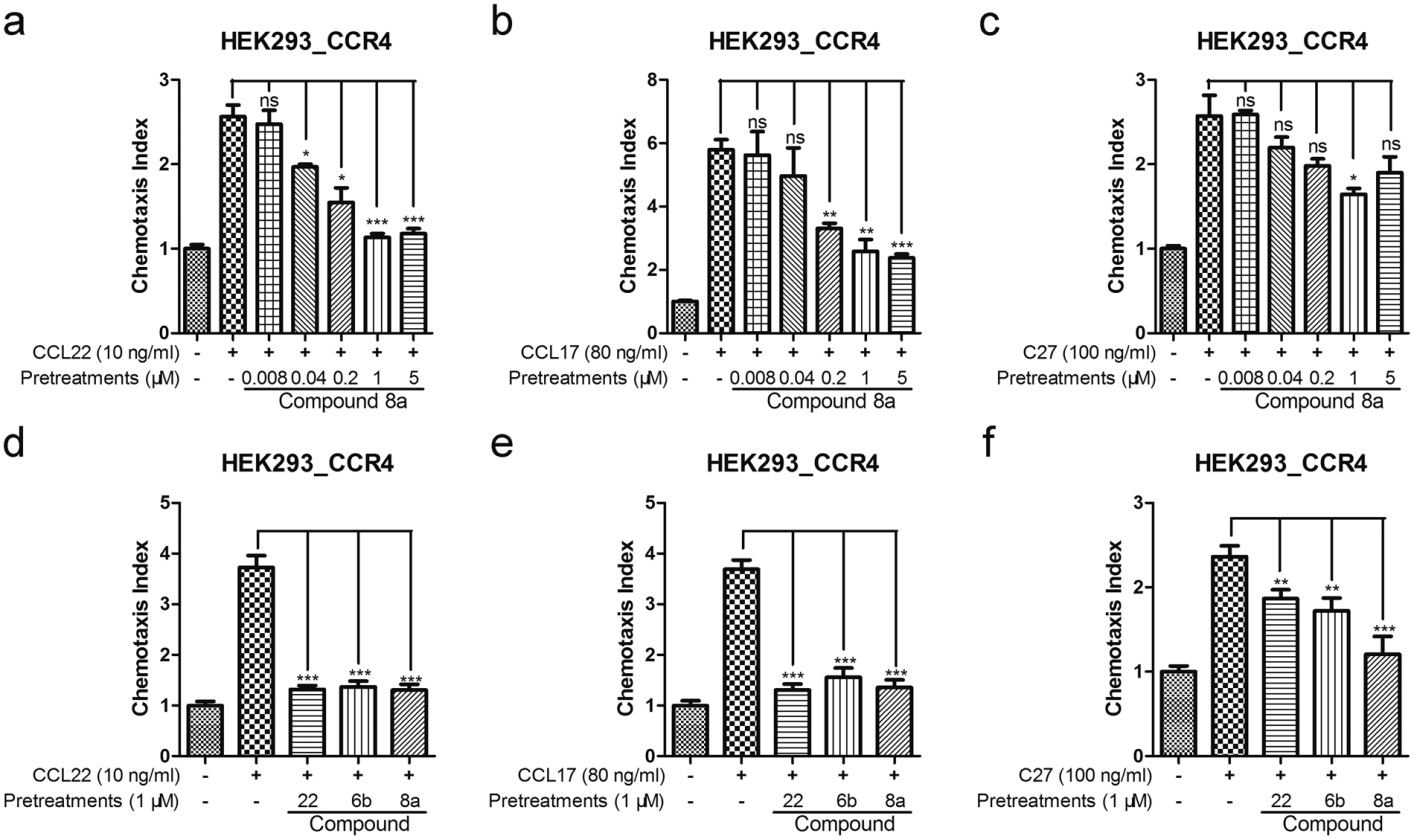
The compound 8a can inhibit the chemotaxis of CCR4-expressing cell lines. (**a**–**c**) In a Boyden chamber system, CCL22 (10 ng/ml), CCL17 (80 ng/ml) and C27 (100 ng/ml) were tested for their ability to chemoattract HEK293-CCR4 cell lines using the methods described in Materials and Methods, pretreated with compound 8a for 30 min. the chemotaxis index and the significant difference compared with the untreated were calculated. (**d**–**f**) CCL22 (10 ng/ml), CCL17 (80 ng/ml)and C27 (100 ng/ml) were tested for their ability to chemoattract HEK293-CCR4 cell lines, pretreated with 1 μM compound 8a, compound 22 and compound 6b for 30 min. The chemotaxis index and the significant difference compared with the untreated were calculated. Error bars show mean ± SEM.*p < 0.05, **p < 0.01, ***p < 0.001.

For identifying the potent compounds (Fig. 1a and b) *in vitro*, we used chemotaxis inhibition assays induced by CCL22, CCL17 and C27. In the chemotaxis inhibition assays, the pcDI-CCR4-transfected HEK293 cells were pretreated with 1 μM compounds respectively before stimulated with CCR4 ligands. As the recombinant protein of CKLF1 is difficult to obtain, we used C27 which also exhibits functional activity via CCR4 instead. At last, we found compound 8a showed excellent potency in blocking the chemotaxis of CCR4 mediated by its three ligands (Fig. 2a,b and c). Compared with compound 22 and compound 6b (a positive CCR4 antagonist derived from compound 22 published before)^25^, compound 8a exhibited a similar significant inhibition of chemotaxis of CCR4 transfected HEK293 to both CCL22 and CCL17, but showed a more positive efficiency in inhibiting of chemotaxis to C27 (Fig. 2d,e and f).

### Specificity of compound 8a

**Figure 3:**
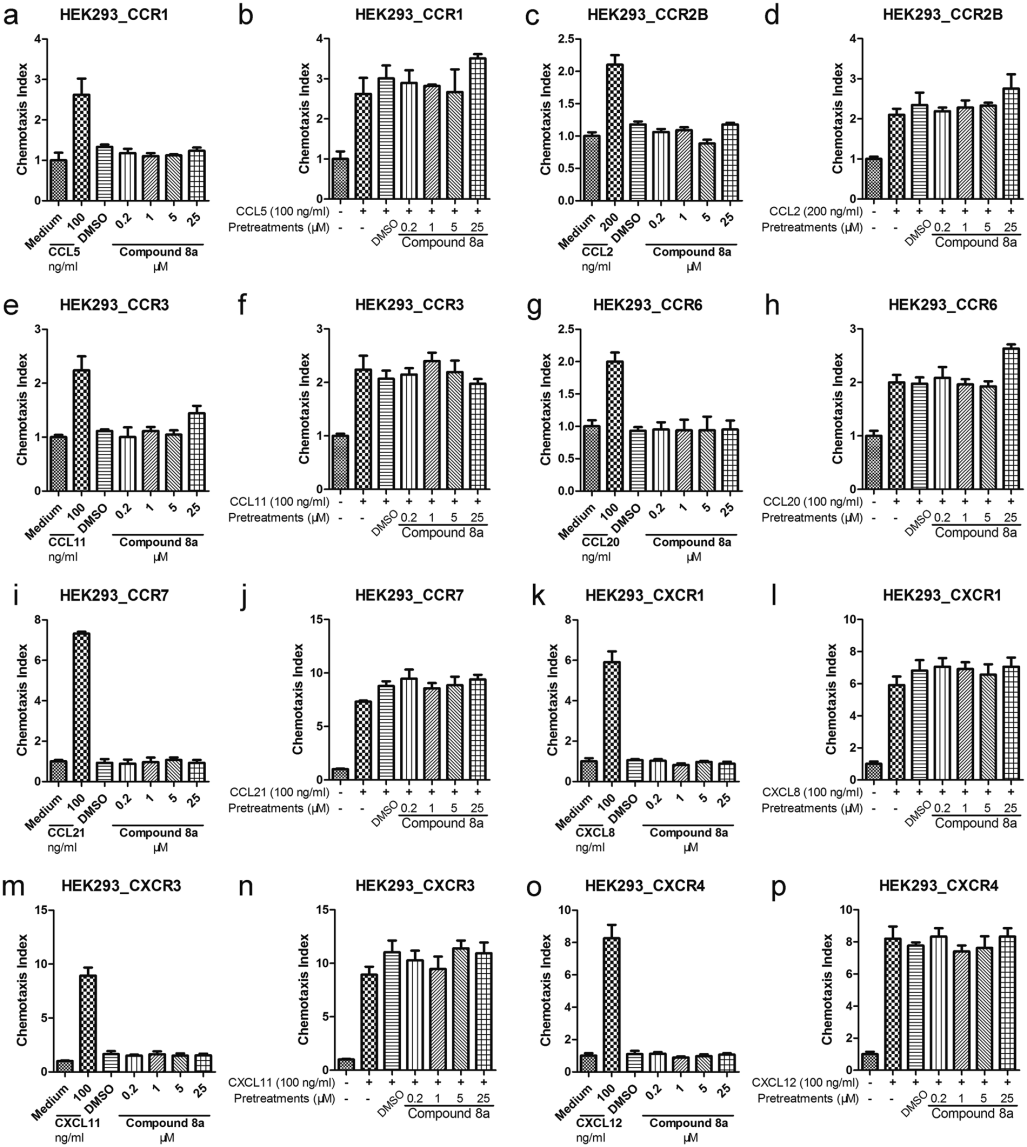
The compound 8a does not have efficacy on other chemokine receptors. The chemotaxis assays and chemotaxis inhibition assays with the HEK293 cells over-expressed different chemokine receptors were used to identify whether the compound 8a had receptor specificity. The compound 8a can specifically inhibit the chemotaxis of CCR4-expressing cell lines. (**a**,**c**,**e**,**g**,**i**,**k**,**m**,**o**) In the chemotaxis assays, compound 8a were tested for their ability to chemoattract HEK293-CCR1, HEK293-CCR2B, HEK293-CCR3, HEK293-CCR6, HEK293-CCR7, HEK293-CXCR1, HEK293-CXCR3 and HEK293-CXCR4 cell lines, respectively. (**b**,**d**,**f**,**h**,**j**,**l**,**n**,**p**) In the chemotaxis inhibition assays, HEK293-CCR1, HEK293-CCR2B, HEK293-CCR3, HEK293-CCR6, HEK293-CCR7, HEK293-CXCR1, HEK293-CXCR3 and HEK293-CXCR4 cell lines were pretreated with a different concentration of compound 8a, respectively.

In order to find out whether the compound 8a had excellent receptor specificity, we used the chemotaxis assays and chemotaxis inhibition assays with the HEK293 cells over-expressed different chemokine receptors. The results showed that compound 8a had an excellent receptor specificity and exhibited few influence on the inhibition of chemotaxis of the HEK 293 cells over-expressed different chemokine receptors to their ligands including CCR1, CCR2B, CCR3, CCR6, CCR7, CXCR1, CXCR3 and CXCR4 (Fig. 3a,c,e,g,i,k,m and o). Moreover, compound 8a didn’t exert chemotaxis effects on the HEK293 cells over-expressed receptors listed before (Fig. 3b,d,f,h,j,l,n and p).

### Affinity between CCR4 and compound 8a

**Figure 4:**
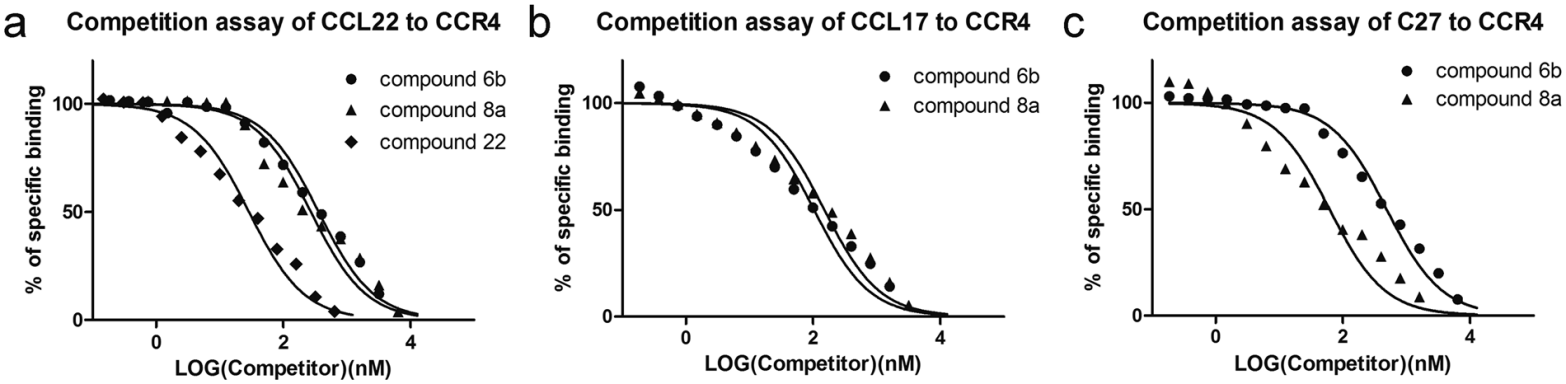
Radio ligand binding assay reveals that compound 8a binds to CCR4 with high affinity. (**a**–**c**) For competitive binding assays, equivalent quantities of cell membrane extract and [^125^I]-CCL22, [^125^I]-CCL17 and [^125^I]-C27 were incubated with varying quantities of unlabeled compound 6b, compound 8a and compound 22, respectively. The y-axis represents the radioactivity of the specific binding complexes, and the x-axis represents the concentration of the competitors.

We further used the binding assay of compound 22^17^, compound 6b and compound 8a to compete CCR4 with CCL22, CCL17 and C27, which are the positive ligands of CCR4. The results showed that in the competitive binding experiments with CCL22 to CCR4, the affinity of compound 22 with CCR4, with IC50 of 28.94 nM, was significantly higher than compound 6b and compound 8a, while the binding of compound 6b (IC50 = 360.2 nM) and compound 8a (IC50 = 272.4 nM) in competition with CCL22 exhibited considerable affinity (Fig. 4a). Moreover, the binding of compound 8a (IC50 = 151.3 nM) in competition with CCL17 is similar to compound 6b (IC50 = 104.1 nM) (Fig. 4b). However, in the competitive binding experiments with C27 to CCR4, compound 8a (IC50 = 61.96 nM) exhibits a higher affinity than compound 6b (IC50 = 489.5 nM)(Fig. 4c).

### Toxicity *invitro* of compound 8a

**Figure 5:**
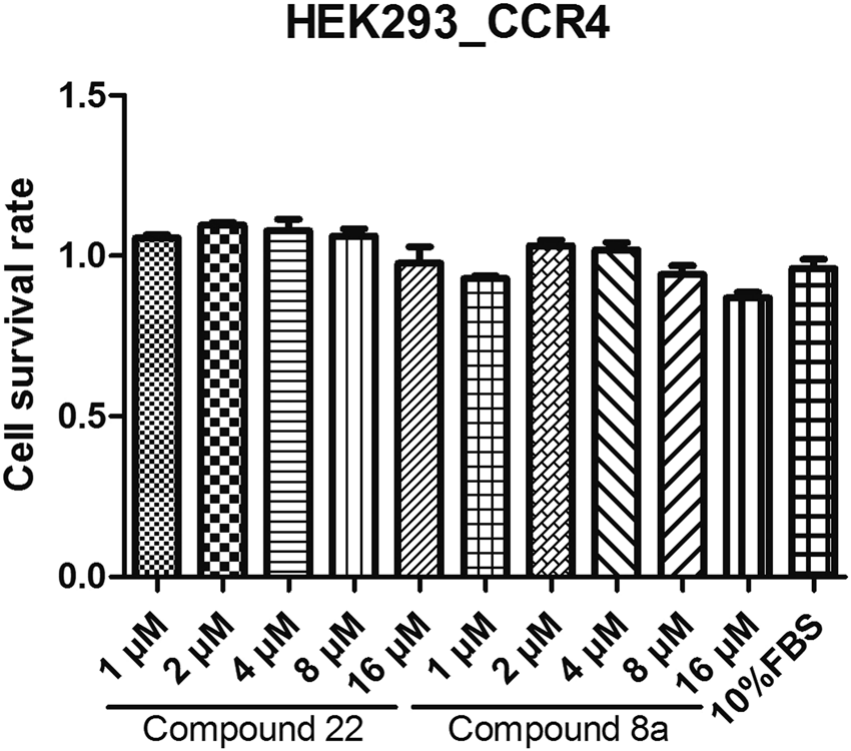
In the MTT assay, 1 μM, 2 μM, 4 μM, 8 μM or 16 μM compound 22 and compound 8a were added in HEK293-CCR4 cell lines for 48 h.

For further studying the toxicity of compound 8a *in vitro*, here we used the 3-(4,5-dimethylthiazol-2-yl)-2,5-diphenyltetrazolium bromide (MTT) assay where HEK293-CCR4 cells were cultured with different concentrations of compound 22 and compound 8a (1 μM, 2 μM, 4 μM, 8 μM and 16 μM). As showed in the results, compound 8a had no obvious cytotoxicity till the higher concentration (16 μM) (Fig. 5).

### Effects of compound 8a on airway hyperresponsiveness (AHR) to aerosolized methacholine (Mch)

**Figure 6:**
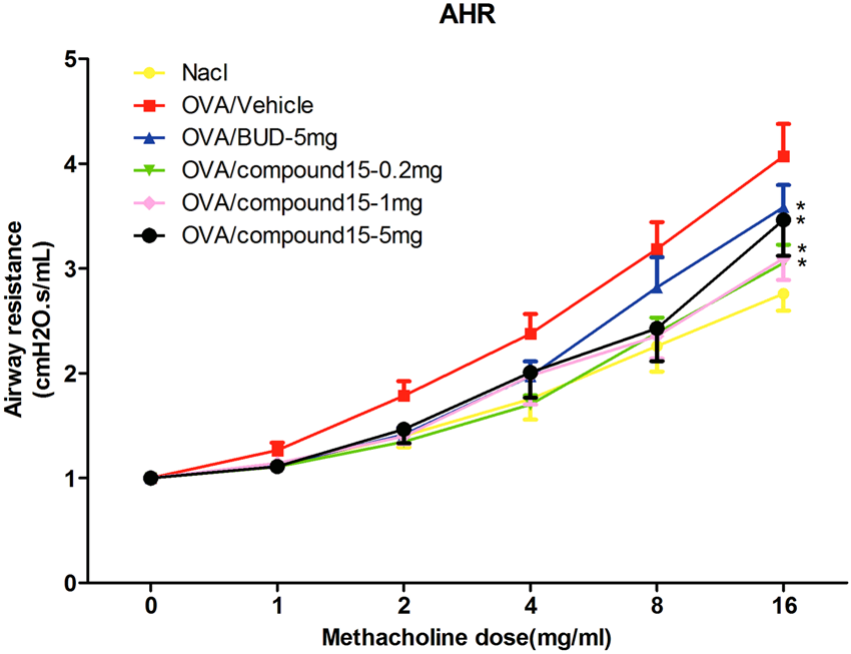
Compound 8a prevented Mch-induced AHR in asthmatic mice. NaCl: NaCl-sensitized and -challenged mice. OVA: OVA-sensitized and -challenged mice. OVA/vehicle: asthmatic mice treated with vehicle. OVA/BUD-5 mg: asthmatic mice treated with 5 mg budesonide. OVA/compound 8a-0.2 mg, 1 mg and 5 mg: asthmatic mice treated with 0.2, 1 or 5 mg compound 8a. n = 6–9 mice per group. Error bars show mean ± SEM. *p < 0.05 by comparison with OVA group treated with vehicle.

To investigate whether compound 8a protects OVA-induced asthmatic mice from AHR, airway responsiveness to increasing dose of methacholine was analyzed 24 h after the final challenge with OVA aerosols. As observed in Fig. 6, airway resistance increases in OVA-sensitized and challenged mice. The mice treated with all doses of compound 8a (0.2 mg, 1 mg and 5 mg) and budesonide (5 mg) have significantly lower AHR to methacholine compared with the asthmatic mice treated with vehicle. Moreover, mice treated with lower doses of compound 8a (0.2 mg and 1 mg) show more effectively alleviated effect on AHR.

### Effects of compound 8a on airway and lung inflammation

**Figure 7:**
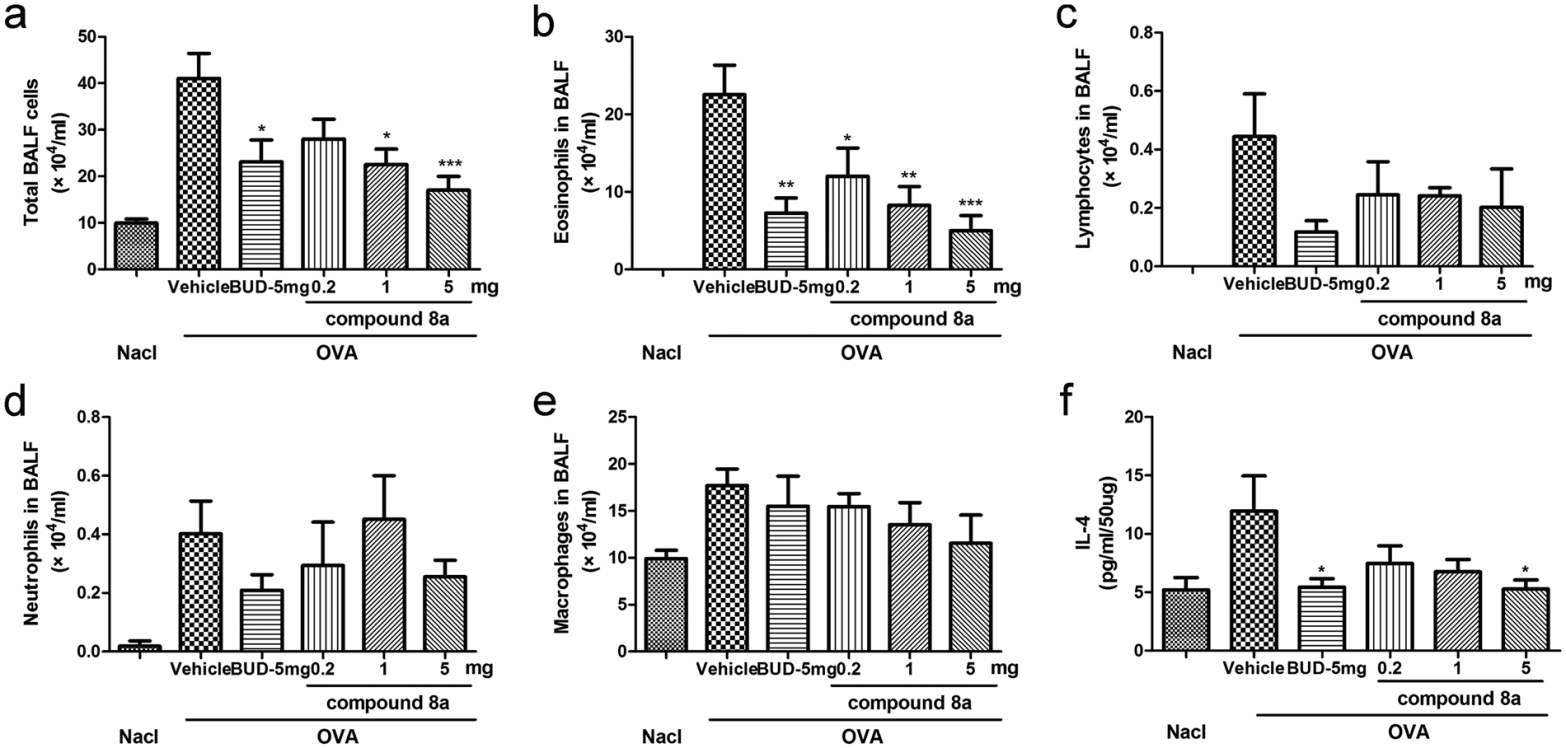
Effect of compound 8a on the number of inflammation cells. (**a**) Total cells in BALF. (**b**) Eosinophils in BALF. (**c**) Lymphocytes in BALF. (**d**) Macrophages in BALF. (**e**) Neutrophils in BALF. (**f**) The concentration of IL-4 in lungs was determined by ELISA. NaCl: 0.9% NaCl-sensitized and -challenged mice. OVA: OVA-sensitized and -challenged mice. OVA/vehicle: asthmatic mice treated with vehicle OVA/BUD-5 mg: asthmatic mice treated with 5 mg budesonide. OVA/compound 8a-0.2 mg, 1 mg and 5 mg: asthmatic mice treated with 0.2, 1 or 5 mg compound 8a. n = 6 mice per group. Error bars show mean ± SEM. *p < 0.05, **p < 0.01, ***p < 0.001 by comparison with OVA group treated with vehicle.

To identify the effect of compound 8a on OVA-induced airway and lung inflammation, we evaluated the bronchoalveolar lavage fluid (BALF) total cell and cell differential counts. Administration of OVA-induced asthmatic mice with compound 8a had a dramatically reduction in inflammatory cell influx in BALF. The total cells count (Fig. 7a) showed that two doses of compound 8a (1 mg and 5 mg) has a remarkably inhibitory effect, which is comparable to budesonide. And the lowest dose of compound 8a (0.2 mg) also reduced the number of total cells, but this effect was marginal. In term of differential leukocyte counts, absolute numbers of eosinophils and lymphocytes significantly decreased in compound 8a (5 mg) or budesonide (5 mg) treated group compared with vehicle treated group (Fig. 7b and c). However, neither compound 8a nor budesonide affect the number of macrophages and neutrophils in BALF (Fig. 7d and e). Therefore, compound 8a mainly showed a benefit effect on OVA-induced airway eosinophilia, which is an important character of asthma.

Additionally, lungs were homogenized for measurement of concentration of Th2 cytokines. The expression of IL-4 was increased following OVA challenge, which was notably decreased after compound 8a (5 mg) or budesonide treatment (Fig. 7f).

**Figure 8:**
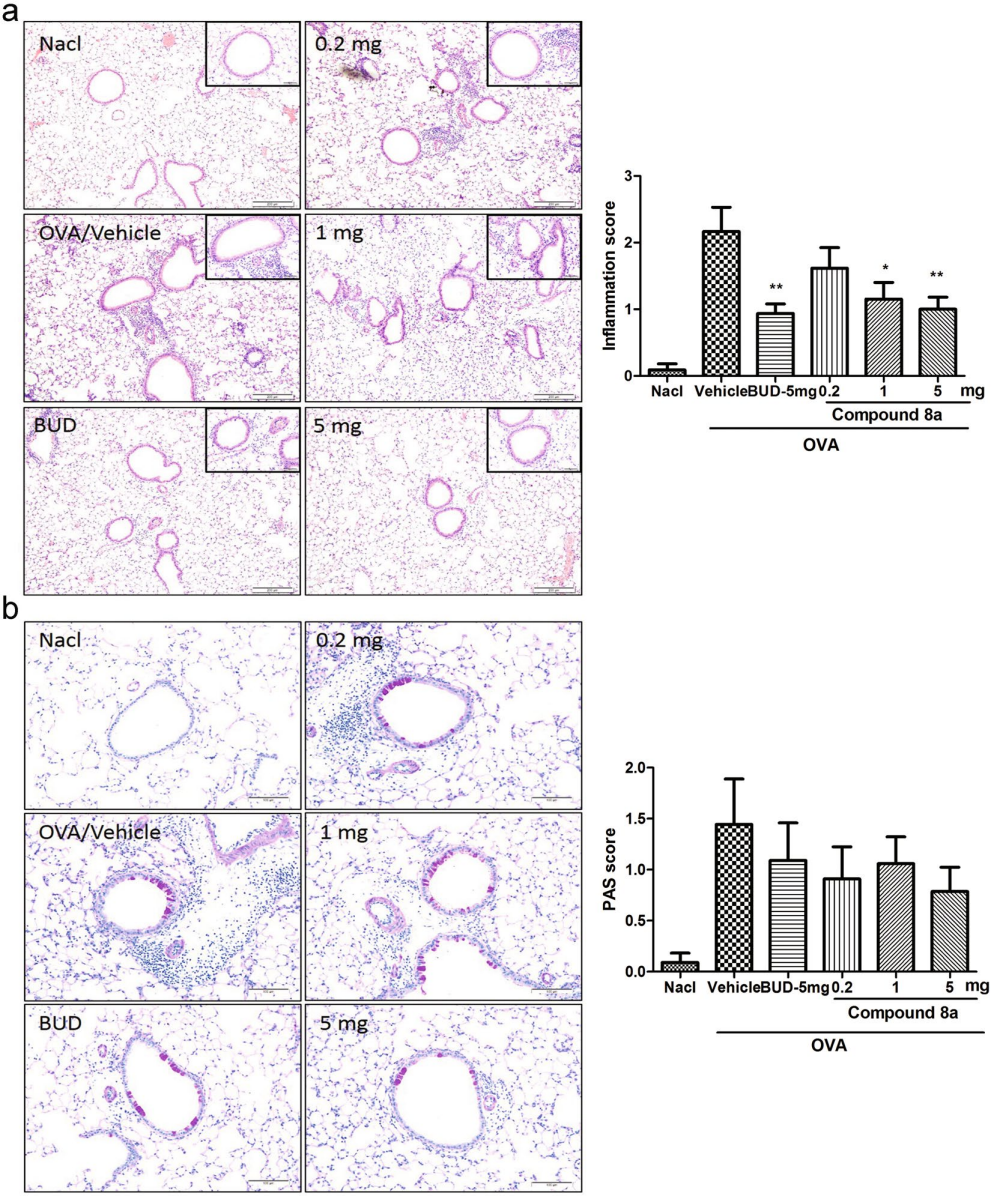
Compound 8a reduced inflammatory infiltrates and goblet cell metaplasia in asthmatic mice. (**a**) Effects of compound 8a on airway and lung inflammation. Scalar bars, 200 μm. (**b**) Effects of compound 8a on airway mucus production. Scalar bars, 100 μm. Lung tissues were stained with hematoxylin/eosin (H&E) or periodic acid-Schiff (AB/PAS), and the stained sections were scored. NaCl: 0.9% NaCl-sensitized and -challenged mice. OVA: OVA-sensitized and -challenged mice. OVA/vehicle: asthmatic mice treated with vehicle of 0.5% DMSO dissolved in the saline. OVA/BUD-5 mg: asthmatic mice treated with 5 mg budesonide. OVA/compound 8a-0.2 mg, 1 mg and 5 mg: asthmatic mice treated with 0.2, 1 or 5 mg compound 8a. n = 6 mice per group. Error bars show mean ± SEM. *p < 0.05, **p < 0.01 by comparison with OVA group treated with vehicle.

To further investigate, lung tissues were collected for histological analysis, which showed that control mice had no inflammation, while OVA sensitized and challenged mice had large number of inflammatory cells infiltrated around the bronchi, and budesonide administration significantly attenuated peribronchial and perivascular inflammation in OVA mice treated with vehicle. Relative to the budesonide, compound 8a (5 mg) also had comparable inhibitory effect (Fig. 8a).

### Effects of compound 8a on airway mucus production

The lung tissues were also assessed for mucus production on airways through Periodic acid-Schiff (PAS) staining. There was an obviously increase in numbers of PAS-stained cells in OVA treated group compared to saline treated mice. Whereas, no difference was observed between vehicle treated and compound 8a treated groups. Budesonide also had no effect on mucus production (Fig. 8b).

## Discussion

Compound 8a is a positive CCR4 antagonist screened from a series of piperazine pyrimidine derivatives, which were designed and synthesized based on the interaction of CCR4 and CKLF1^25^. For identifying the new designed compounds *in* *vitro*, we used chemotaxis inhibition assays induced by the three positive ligands of CCR4 (CCL22, CCL17 and one of CKLF1’s C-terminal peptides, C27). We found that compound 8a showed obvious inhibitory effect in chemotaxis assays induced by the three positive ligands of CCR4. Compared with the known compound 22 and compound 6b, compound 8a has higher chemotaxis inhibition rate in the chemotaxis assays induced by C27.

For studying the receptor specificity of compound 8a, we adopted chemotaxis and chemotaxis inhibition assays with HEK293 cells that overexpressed different chemokine receptors. We chose CCR1-7 which were the same chemokine receptor family of CCR4, and CXCR1, CXCR3 and CXCR4 that were high expressed in immune cells. The results showed that compound 8a exerted few influence on the interaction of other receptors and their ligands even at a higher concentration (25 μM). Moreover, compound 8a didn’t show chemotaxis effects on the cells over-expressed these receptors. This part proved that compound 8a had excellent receptor specificity *in* *vitro*.

We used MTT assays to analyze the cytotoxicity of compound 8a. The results showed that compound 8a couldn’t affect the cell vitality till the higher concentration (16 μM).

The study of the compound 8a efficacy *in* *vivo* demonstrated that CCR4 blockade by compound 8a effectively attenuate AHR, airway eosinophilia, and Th2 cytokines in a mouse model of OVA-induced asthma.

Asthma is a Th2-dominant disease. Th2 cells are recruited into airway after allergens challenge, and play as central orchestrators of allergic airway inflammation in asthma by producing Th2 cytokines. Among Th2 cytokines, IL-4 and IL-13 exhibit partly functional overlap due to combination with IL-4Rα. IL-4 has been proved to promote recruitment of eosinophils and production of IgE by B cells^26^. In our study, among the three doses, the high dose of compound 8a (5 mg) obviously reduced the expression of IL-4, contributing to the best protective effect on airway eosinophilia and trafficking of activated T cell into airway in asthmatic mice.

Th2 cells are the primary drivers of mild to moderate and allergic asthma. The accumulation of Th2 T cells in the lungs is essential for both the initiation and persistence of airway inflammation, and studies in asthmatic volunteers have shown marked increases in Th2 T cells in the lungs after *in* *vivo* allergen challenge^2–5,27^. CCR4 has been long thought to take part in the recruitment of Th2 cells following allergen exposure, owing to its high expression on Th2 cells.

It is well known that the CCR4 and its ligands CCL17 and CCL22 played an important role in allergic diseases. In asthmatic humans, the number of CCR4-expression T cells was increased, and the expression of CCL17 and CCL22 was also upregulated in the airway upon allergen challenge^9,27^. Chemokine-like factor 1 (CKLF1) also uses CCR4 as functional receptor^12^. CKLF1 is highly expressed on the bronchial mucous membrane of asthma patients. Mice with overexpressed CKLF1 have significant pathological changes that are similar to those of asthma, such as airway remodeling, peribronchial leukocyte infiltration in addition to epithelial shedding, collagen deposition, inflammatory exudates in the lumen^14^. Similar and obvious changes were also taken place in the lungs of CKLF1-transgenetic mice (unpublished data). More recently, in studies blocking CCR4/CCL17/CCL22 axes with antibodies or small molecule antagonists was found to success in inhibiting several important features of allergic airways inflammation, including airway eosinophilia, bronchial hyperreactivity, goblet cell proliferation^11,28,29^. These studies implicate that the CCR4/CCL17/CCL22 axes could be considered as targets for effective therapy of bronchial asthma. In this study, compound 8a not only efficiently inhibited the interaction of CCL22 or CCL17 with CCR4, but also showed a more positively antagonistic action of C27.

In conclusion, the data from this study demonstrate for the first time that compound 8a as a small molecule antagonist specifically targeting CCR4/CCL17/CCL22/CKLF1 axes effectively attenuates the allergic airways inflammation in a murine model of allergic asthma *in vivo*. CCR4 blockaded by compound 8a effectively inhibits Th2 cytokines and consequent airway eosinophilia and hyperresponsiveness. Therefore, compound 8a may serve as a novel therapeutic strategy in the treatment of asthma.

## Methods

### Synthesis of Compound 8a

{4-[4-Chloro-6-(2,4-dichloro-benzylamino)-pyrimidin-2-yl]-piperazin-1-yl}-piperidin-2-yl-methanone. Compound 8a (structure is shown in Fig. 1) was synthesized according to a well-established literature procedure^17^. m. p. > 220 °C. ^1^H-NMR (400 MHz, dimethyl sulfoxide (DMSO)) δ ppm: 9.13 (1 H, s), 8.63 (1 H, s), 8.08 (1 H, s), 7.64 (1 H, d, J = 1.52 Hz), 7.27 (2 H, m), 5.97 (1 H, s), 4.56 (2 H, d, J = 4.48 Hz), 4.39 (1 H, d, J = 9.24 Hz), 3.76-3.22 (9 H, brm), 2.87 (1 H, s), 1.99 (1 H, m), 1.74 (4 H, m), 1.48 (1 H,m); EI-MS (m/z): 483.2 [M +  H]^+^.

### Chemokines

CCL2, CCL5, CCL11, CCL17, CCL20, CCL21, CCL22, CXCL8, CXCL11 and CXCL12 were purchased from PeproTech.

### Chemokine receptors

The pcDB-CCR2B (NM_001123041), pcDI-CCR4 (NM_005508), pcDI-CCR5 (NM_000579), pcDI-CCR6 (NM_004367), and pcDB-CCR7 (NM_001838) plasmids were constructed in our laboratory. The cDNA fragments of human CCR2b, CCR4, CCR5, CCR6, and CCR7 were cloned from the first chain of PBMC cDNA library by RT–PCR technique. The cDNA fragments of the receptors were inserted into the pGEM-T Easy Vector (Promega), respectively. Then they were respectively inserted into the pcDI (constructed in our laboratory) and pcDNA3.1. CXCR3 (NM_001142797) and CXCR4 (NM_001008540) expression plasmids were provided by Chinese National Human Genome Center. CCR1 (NM_001295), CCR3 (NM_001164680), and CXCR1 (NM_000634) expression plasmids were kindly provided by Dr. Philip M. Murphy (Laboratory of Molecular Immunology, National Institute of Allergy and Infectious Diseases, National Institutes of Health, Bethesda, MD). CCR2A and CCR2B share the same N-terminal sequence and distribution^13^, but the expression level of CCR2B is 9-fold higher than that of CCR2A on cell membranes^14^.

### Cell culture

HEK293 cells (The American Type Culture Collection (ATCC)) were cultured according to ATCC recommendations.

### Radio ligand binding assay

A HEK293 cell line stably expressing CCR4-enhanced green fluorescent protein was established. CCL22, CCL17 and C27 were labeled with [^125^I]. For competition binding assays, equivalent quantities of cell membrane extract and [^125^I]-CCL22, [^125^I]-CCL17 and [^125^I]-C27 were incubated with varying quantities of unlabeled compound 8a, compound 6b and compound 22. All reactions were incubated at 4 °C for 24 h. After the binding step, 25% polyethylene glycol (6000–8000) and 0.5% human g-globulin were added, and the mixture was incubated at room temperature for 15 min. Binding complexes were obtained by centrifuging the mixture at 3500 rpm for 20 min at 4 °C, and radioactivity was evaluated. Results were corrected for “blank” background radiation, defined as the radioactivity precipitated by polyethylene glycol–g-globulin from the same amount of radioactive ligand in binding buffer in the absence of cells.

### Chemotaxis inhibition assay

Chemokine receptor expression plasmid was transiently transfected into HEK293 by electroporation at 120 V for 20 ms, using an electric pulse generator (Electro Square Porator ECM 830; BTX, San Diego, CA). Forty-eight-well micro chemotaxis chambers (Neutroprobe, Bethesda, MD, USA) were used in this assay. Forty-eight hours later, the cells were pretreated with compounds or compounds dissolved medium (1‰ DMSO) for 30 min at 37 °C prior to stimulation with chemoattractant. Chemoattractant diluted in RPMI 1640 medium supplemented with 0.1% bovine serum albumin (BSA) was placed in the lower chambers (27.5 μl/well). In these assays, CCL22 and CCL17 (Peprotech, Rocky Hill, NJ, USA) were used as chemoattractant and the final concentrations of chemoattractant were 10 ng/ml and 80 ng/ml respectively. The pcDI-CCR4-transfected HEK293 cells were resuspended in the same medium at a density of 1 × 10^6^ cells/ml and added to the upper chambers (55 μl/well). The upper and lower chambers were separated by a Rat Tail Collagen Type 1 (Biomedical Technologies, Stoughton, MA, USA) coated polyvinylpyrrolidone-free polycarbonate membranes (Neuro Probe) with 12-μm pores. The chambers were incubated for 6 h at 37 °C in an atmosphere of 5% CO2 and 95% air. Afterward, cells migrated to the lower part of the filter. The membranes were removed from the chambers, washed, fixed and stained with the Three Step Stain Set (Richard-Allen Scientific Michigan, MI, USA). Cells that migrated into each filter were counted in five randomly selected high-power fields (400 × ) per well. All samples were assayed at least three times. The chemotactic index was calculated and expressed as the number of cells that migrated to the sample over the number of cells that migrated to the control medium.

### Chemotaxis assays for receptor specificity of compounds

HEK293 cells were transfected with different chemokine receptors. Compound 8a with DMSO was diluted to 0.2 mM, 1 mM, 5 mM and 25 mM, and then we used RPMI 1640 medium containing 0.1% BSA for dilution. The final concentration is: 0.2 μM, 1 μM, 5 μM and 25 μM. DMSO was also diluted 1000-fold with 0.1% BSA RPMI 1640 medium. Chemokines was also diluted to an appropriate concentration. The dilutions of Compound 8a, 1‰ DMSO, the corresponding chemokines of different receptors and 0.1% BSA RPMI 1640 medium were placed in the lower chambers (27.5 μl/well). The transfected HEK293 cells were resuspended in the same medium at a density of 1 × 10^6^ cells/ml and added to the upper chambers (55 μl/well).

### Chemotaxis inhibition assays for receptor specificity of compounds

HEK293 cells were transfected with different chemokine receptors. The corresponding chemokines of different receptors were placed in the lower chambers. The transfected cells were pretreated with different concentrations of Compound 8a or 1‰ DMSO for 30 min at a density of 1 × 10^6^ cells/ml and added to the upper chambers, the final concentration of the compound is: 0.2 μM, 1 μM, 5 μM, and 25 μM, respectively.

### MTT assay

pcDI-CCR4-transfected HEK293 cells, at the density of 800 cells /well, were plated overnight in 96-well plates. After plated overnight, different concentrations of compound 22 and compound 8a were added in cells and the final concentrations of these two compounds were: 1 μM, 2 μM, 4 μM, 8 μM and 16 μM. We set four wells for cells in each group which were incubated with each concentration of the compounds, and the cells were cultured for 48 h or 72 h. At 48 h or 72 h, 10 μl of the 5 mM MTT were added in cells and 100 μl of cell lysis buffer were added 4–6 h later. After added cell lysis buffer for an hour, microplate autoreader were used for measuring optical density (OD) of cells at 570 nm, and the ratio of OD in each group and that of DMSO group were valued as cell survival rates of each group.

### Mice

Male BALB/c 6 week old mice were purchased from the Experimental Animal Center of Zhejiang University. All mice were raised in a specific pathogen-free animal facility. All animal experiments were carried out according to the guidelines for the care and use of laboratory animals and were approved by the ethics committee of Zhejiang University and Peking University, China.

### Generation of allergic asthma

Mice were sensitized and challenged with chicken egg OVA (sigma-Aldrich, St Louis, MO, USA) as described in details previously^16^. Briefly, after sensitized by intraperitoneal injection of 100 μl (80 μg) OVA in 100 μl alum (Pierce, Rockford, IL, USA) on days 0 and 14, mice were exposure to 1% OVA aerosols for 40 min on days 24–26. Control mice were injected intraperitoneally with saline and exposed to saline aerosols.

### Injection of compound 8a

Compound 8a was dissolved in saline with DMSO (0.5%). Half an hour before each OVA challenge, the groups of mice treated with various doses of compound 8a (0.2, 1, 5 mg) inhaled aerosolized compound 8a for 45 min on days 24–26. Compound 8a aerosols were produced by using a Plexiglas chamber and ultrasonic nebulizer. While the budesonide (BUD) group were administered with 5 mg budesonide, and the vehicle group were treated with compound-dissolved vehicle. The Saline was treated as the control.

### Airway responsiveness

Twenty-four hours after the last challenge, mice were anesthetized and tracheotomized with a cannula, then ventilated at a fixed breathing rate. Once being stabilized, mice were challenged via the airways with saline or increasing concentrations of Mch (1, 2, 4, 8, 16 mg/ml) by nebulization. Airway resistance was measured by FinePointe Series RC Sites (Buxco Research System). Changes in airway resistance were calculated for each Mch concentration.

### Bronchoalveolar Lavage

Twenty-four hours after the last exposure, collection of BALF and cell count was performed as previously described. Briefly, lavage was performed by instilling 0.4 ml PBS into lungs and with drawn to collect the cells three times. Total BALF cells were counted. After be centrifuged, the remaining cells was spun onto glass slides, and stained with Wright–Giemsa stain. Differential counts were evaluated by counting 400 total cells.

### Enzyme linked immunosorbent assay (ELISA)

Twenty-four hours after the last challenge, lungs were removed from control or asthmatic mice, and they were cut into pieces and homogenized in 500 μl PBS, centrifuged (2000 rpm, 10 min) and the supernatant collected. The concentration of IL-4 in lungs were determined by ELISA kit (Sensitivity: 2 pg/ml, R&D Systems, Minneap-olis, MN, USA) following the manufacturer’s protocol.

### Histological examination

After sacrificed, the lungs were fixed in formalin overnight. Then they were embedded in paraffin, cut into sections, and stained with hematoxylin/eosin (H&E) or periodic acid-Schiff (PAS) following standard protocol. Inflammation was scored according to published guidelines^30^. And PAS-stained goblet cells in airway epithelium were quantified as described previously.

## Statistical analysis

Results are presented as means ± SEM. Data were analyzed with GraphPad Prism 5.01 (GraphPad Software) using Student’s *t*-test. A *p* value less than 0.05 was considered to be statistically significant.
