# Publisher Correction: A new antagonist for CCR4 attenuates allergic lung inflammation in a mouse model of asthma

**DOI:** 10.1038/s41598-018-23185-w

**Published:** 2018-04-26

**Authors:** Yang Zhang, Yinfang Wu, Hui Qi, Junhai Xiao, Hongwei Gong, Yan Zhang, Enquan Xu, Song Li, Dalong Ma, Ying Wang, Wen Li, Huahao Shen

**Affiliations:** 1 Department of Immunology, School of Basic Medical Sciences, Key Laboratory of Immunology, Ministry of Health, Peking University Health Science Center, Beijing, 100191 China; 2 Laboratory of Computer-Aided Drug Design & Discovery, State Key Laboratory of Toxicology and Medical Countermeasures, Beijing Institute of Pharmacology and Toxicology, Beijing, 100850 China; 3 Department of Respiratory Medicine, the Second Affiliated Hospital School of Medicine of Zhejiang University, Zhejiang University institute of Respiratory Diseases, Hangzhou, 310009 China; 4 School of Pharmaceutical Sciences, Jilin University, Changchun, 130021 China; 5 Department of Hematology, Peking University First Hospital, Beijing, 100034 China; 6 Beijing Children’s Hospital, Capital Medical University, Beijing, 100045 China

Correction to: *Scientific Reports* 10.1038/s41598-017-11868-9, published online 08 November 2017

The original version of this Article contained an error in the order of author names, which were incorrectly given as ‘Yang Zhang, Yinfang Wu, Hui Qi, Hongwei Gong, Yan Zhang, Enquan Xu, Song Li, Dalong Ma, Junhai Xiao, Ying Wang, Wen Li & Huahao Shen’.

In addition, the version of this Article previously published omitted Junhai Xiao as an equally contributing author.

These errors have now been corrected in the HTML and PDF versions of this Article.

